# Supplementary material for: Drug repurposing for aging research using model organisms
Source: Aging Cell. 2017 Jun 16;16(5):1006–15. doi: 10.1111/acel.12626 (PMC5595691; doi:10.1111/acel.12626)
Supplement: Supplementary file 7 — Data S1 Zip‐Archive of all report cards. [file ACEL-16-1006-s007.zip › RC_OQB.pdf]

OQB

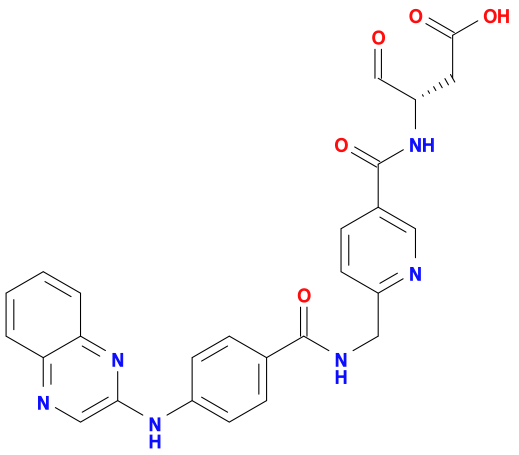

Database identifiers

ChEMBLCompound	CHEMBL202160

Ranking

	Rank	Score
Drosophila	445/697	0.323
C. elegans	471/591	0.098

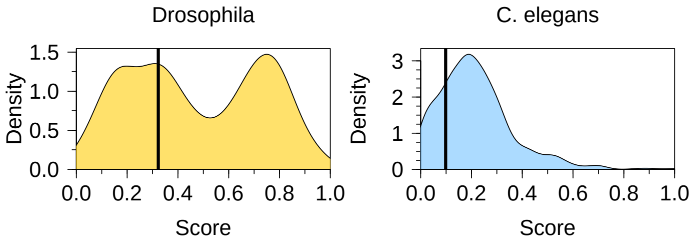

	Ageing implication	Domain conservation	Binding site conservation	Binding affinity	Bioavailability	Lipinski	Promiscuity	Purchasability	Drug approval	**Total**
Drosophila	1.0	0.687	0.595	0.878	(0.9)	0.0	-0.0	0.0	0.0	0.323
C. elegans	1.0	0.589	0.66	0.878	0.29	0.0	-0.0	0.0	0.0	0.098

Names

- (3S)-4-keto-3-6-4-(quinoxalin-2-ylamino)benzoylaminomethylpyridine-3-carbonylaminobutyric acid
- (3S)-4-oxo-3-6-4-(quinoxalin-2-ylamino)benzoylaminomethylpyridine-3-carbonylaminobutanoic acid
- (3S)-4-oxo-3-6-4-(quinoxalin-2-ylamino)phenylcarbonylaminomethylpyridin-3-ylcarbonylaminobutanoic acid
- (3S)-4-oxo-3-oxo-6-oxo-4-(2-quinoxalinylamino)phenylmethylaminomethyl-3-pyridylmethylaminobutanoic acid
- 4-oxo-3-(6-4-(quinoxalin-2-ylamino)-benzoylamino-methyl-pyridine-3-carbonyl)-amino-butyric acid

Roles

ChEBI entry None has no roles

Status

Approved drug (according to ChEMBL)	No
Number of Rule of 5 violations	0
Binding affinity to original target in log units (RF-Score prediction)	6.97
Burns *C. elegans* bioavailability prediction	-0.66

Compound Target Characteristics

Caspase-1

Best gene implication in ageing for this target family came from gene Q9XYF4 annotated in UniProt release 2014_02. Annotation GO 8340 (determination of adult lifespan) was Inferred from Mutant Phenotype

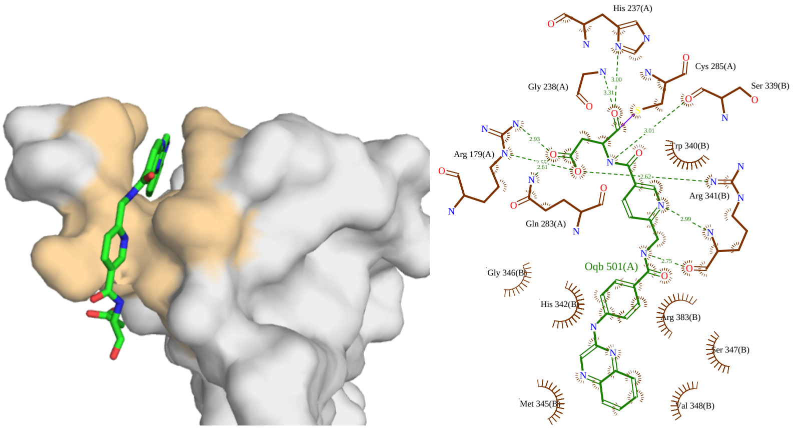

protein	amino acids contacts (binding site)													
PDB:1rww:chainA:P29466	R	H	G	Q	C	-	-	-	-	-	-	-	-	-
PDB:1rww:chainB:P29466	-	-	-	-	-	S	W	R	H	M	G	S	V	R
tr:G3V169:G3V169_HUMAN	R	H	G	Q	C	S	W	R	H	M	G	S	V	R
sp:P29466:CASP1_HUMAN	R	H	G	Q	C	S	W	R	H	M	G	S	V	R
tr:F1LQZ4:F1LQZ4_RAT	R	H	G	Q	C	S	W	R	H	Q	G	S	L	R
sp:P29452:CASP1_MOUSE	R	H	G	Q	C	S	W	R	H	R	G	S	L	R
sp:Q9XYF4:ICENC_DROME	R	H	G	P	C	T	H	R	D	T	G	S	W	S
tr:G5EG94:G5EG94_CAEEL	R	H	G	S	C	S	Y	R	D	A	G	T	W	V
tr:G5EBM1:G5EBM1_CAEEL	R	H	G	S	C	S	Y	R	D	A	G	T	W	V

protein	whole protein ident	whole protein simil	domain-based ident	domain-based simil	contact-based ident	contact-based simil
PDB:1rww:chainA:P29466	1.0	1.0	1.0	1.0	0.36	0.0
PDB:1rww:chainB:P29466	1.0	1.0	1.0	1.0	0.64	0.29
tr:G3V169:G3V169_HUMAN	0.91	0.91	1.0	1.0	1.0	1.0
sp:P29466:CASP1_HUMAN	1.0	1.0	1.0	1.0	1.0	1.0
tr:F1LQZ4:F1LQZ4_RAT	0.61	0.86	0.69	0.9	0.86	0.91
sp:P29452:CASP1_MOUSE	0.62	0.86	0.67	0.89	0.86	0.92
sp:Q9XYF4:ICENC_DROME	0.16	0.62	0.22	0.68	0.5	0.6
tr:G5EG94:G5EG94_CAEEL	0.13	0.42	0.2	0.61	0.5	0.66
tr:G5EBM1:G5EBM1_CAEEL	0.11	0.47	0.2	0.64	0.5	0.66

Nc (FBgn0026404) associated phenotypes

cell death defective, cell shape defective, chemical resistant, decreased cell death, decreased cell number, developmental rate defective, increased cell death, increased cell number, lethal - all die before end of P-stage, lethal - all die before end of first instar larval stage, lethal - all die before end of pupal stage, maternal effect, melanotic necrosis, neuroanatomy defective, partially, partially lethal - majority die, rescuable maternal effect, short lived, size defective, somatic clone, some die

during first instar larval stage, some die during pharate adult stage, some die during pupal stage, temperature conditional

(Information from FlyBase)

Nc (UniProt:Q9XYF4) annotation

Function: Involved in the activation cascade of caspases responsible for apoptosis execution. Effector of steroid-mediated apoptosis during insect metamorphosis. Overexpression promotes programmed cell death. Interaction with th is required to suppress Nc-mediated cell death; via th-mediated ubiquitination of Nc. Rate-limiting caspase in rpr and W death pathway. (PubMed:10200258, PubMed:10675329, PubMed:10984473, PubMed:14517550).

Subunit: Interacts with th; residues 114-125 interact with the second BIR domain of th. Can form a stable complex with Ice. Rpr can out-compete Nc for binding th, therefore removing th-mediated ubiquitination. (PubMed:10675329, PubMed:14517550).

Subcellular location: Cytoplasm ECO:0000250.

Tissue specificity: Ubiquitously expressed in embryos during early stages of development. In late third instar larvae, dramatic up- regulation in salivary glands and midgut before histolysis of these tissues. (PubMed:10200258).

Developmental stage: Expressed both maternally and zygotically. (PubMed:10200258).

Induction: By ecdysone; exposure of salivary glands and midgut isolated from second instar larvae results in a massive increase in levels. (PubMed:10200258).

Ptm: Ubiquitinated by th, leading to its subsequent degradation. (PubMed:12021771).

Miscellaneous: The promiscuous caspase inhibitor p35 is neither cleaved by Nc in vitro nor blocks Nc activity in vivo. (PubMed:10675329).

(Information from UniProt)
